# The motivational drives of sickness: Acute changes in self-rated motivation during experimental endotoxemia assessed with the newly developed Motivation Scale of Sickness (MOSSick)

**DOI:** 10.1016/j.cpnec.2025.100327

**Published:** 2025-11-13

**Authors:** Rasmus Skarp, Lina S. Hansson, Tina Sundelin, Sofie Paues, Martin Janson, Leonie JT. Balter, Mats J. Olsson, John Axelsson, Mats Lekander, Julie Lasselin

**Affiliations:** a Stockholm University, Department of Psychology, Stockholm, Sweden; b Karolinska Institutet, Department of Clinical Neuroscience, Division of Psychology, Solna, Sweden; c Osher Center for Integrative Health, Department of Clinical Neuroscience, Karolinska Institutet, Stockholm, Sweden; d Department of Clinical Sciences, Karolinska Institutet, Stockholm, Sweden; e Department of Anaesthesia and Intensive Care, Danderyd Hospital, Stockholm, Sweden; f Department of Psychiatry, Radboud University Nijmegen Medical Centre, Nijmegen, Netherlands; g Donders Institute for Brain, Cognition and Behavior, Radboud University Nijmegen, Nijmegen, Netherlands; h Department of Biomedical and Clinical Sciences, Linköping University, Sweden

**Keywords:** Sickness behavior, Motivation, Inflammation, Lipopolysaccharide, Physical activity, Care seeking

## Abstract

While altered motivation is central in sickness behavior, previous research has mainly focused on motivation for rewards, rather than motivational changes in a broader perspective. In a larger study following a randomized within-subject placebo-controlled crossover design, we investigated the effects of systemic inflammatory activation on self-rated motivation in 21 healthy participants, using an intravenous injection of 2.0 ng/kg body weight lipopolysaccharide (LPS) compared to an intravenous injection of saline (placebo). Self-rated motivation was measured before, 3 h, and 7.5 h post-injection using the Motivation Scale of Sickness (MOSSick), a newly developed tool designed to assess motivational changes during sickness. It contains 26 items covering motivational drives related to hunger, thirst, and food preferences; resting, physical, and social activities; care seeking; and utilization of resources (i.e., willingness to ‘pay’ and ‘walk’) to be able to rest or to be healthy at once. At the peak of the sickness response (3 h post-LPS injection), there was an increased motivation to seek care, rest, and sleep, as compared to placebo, while motivation to partake in physical and social activities decreased. Several of these effects remained at 7.5 h post-injection. When in the LPS condition, participants were also willing to pay more money to rest and to be healthy compared to when in the placebo condition. Hunger increased over time in both conditions, but less after LPS administration. During the peak of the sickness response, higher sickness ratings were weakly associated with lower motivation for social activities and higher motivation for resting and sleeping. No further association was found between motivational drives and sickness ratings or other sickness measures, i.e. concentrations of cytokines and tympanic temperature, although sample size was limited for these analyses. These findings illustrate that motivational changes during acute sickness are not restricted to a general decrease in motivation. Instead, sick individuals are more motivated to take part in behaviors that enable energy preservation, care, and recovery, compared to when healthy.

## Highlights

• The study examined motivational changes during sickness using the MOSSick questionnaire.
• Motivation was assessed after LPS administration in a randomized placebo-controlled design
• LPS increased drive to rest, sleep, and seek care, while reducing social and physical activity.
• Motivation to pay for rest or health was higher after LPS than placebo.
• Appetite increased overall, but less so after LPS.

## 1 Introduction

The sickness response involves a plethora of behavioral changes, including reduced social and physical activity, decreased food intake, and attentional deficits [1,2]. This so-called *sickness behavior* is induced by proinflammatory cytokines produced during activation of the immune system and acting on the brain to modulate brain functions [1,3]. Sickness behavior represents a well-established phenomenon prevalent across species [4]. Sickness behavior is viewed as adaptive, as it helps organisms conserve the energy required for supporting immune cell functions and the mounting of fever, which are highly energy-demanding [1,2,5,6]. In order to preserve body energy, a reorganization of motivational priorities is required [5]. For example, motivated behaviors such as locomotion and engagement in social behaviors are clearly reduced in sick animals [[1], [2], [3],^7^]. In spite of the substantial behavioral and emotional changes that follow immune activation along with early observations and theories focusing on the role of motivation in sick animals [2,7,8], motivational changes remain poorly characterized in humans. A common simplification seems to be that motivation is reduced rather than selectively altered during sickness, in itself representing motivated behavior. Most of the research on inflammation-induced reorganization of motivational priorities in humans has focused on sensitivity to reward and punishment [9], effort expenditure for rewards [[10], [11], [12]] and related neural activity [9,13], or been inferred from animal behaviors [7,8,[14], [15]].

Prior research has demonstrated that external demands can moderate motivated behavior during sickness. For example, the pioneering studies by Aubert et al [7,8] found that, during sickness, dams mice build more robust nests and retrieve their pups more if ambient temperature is lowered to 6 °C, as compared to in normal room temperature. It has also been found that sick mice, as compared to healthy mice, show a proportionally greater reduction in effort exerted for low-effort grain rewards as compared to high-effort chocolate rewards [14]. A similar pattern has been observed in human studies using monetary rewards, with sick people exhibiting increased willingness to exert effort in button presses in order to get a monetary reward [10,12]. However, contradicting results have been found when measuring effort through squeeze strength, and reward motivation through willingness to accept differing effort and reward options. Specifically, high-effort options were instead accepted less often than low-effort options during sickness [11]. Differences in task settings could explain these results: in the former study, participants had to choose between a high-effort and a low-effort option, whereas in the latter, they chose between making an effort or doing nothing. Furthermore, other contextual factors, such as the social and cultural context are also likely to play a role in motivation during sickness [16,17]. Notably, reduced motivation to exert *mental* effort has also been observed during sickness, indicating that sickness influences effort allocation more broadly and not only in the physical domain [18]. A recent experimental study has found that low-grade inflammation was associated with an increased drive towards rest and sleep, and avoidance of physical and social behavior [19]. However, individuals in that study were not particularly sick, and the questionnaire was limited with regard to items of motivations towards seeking care at a hospital and other sickness motivation behavior. Thus, it seems important to characterize the various components of motivational changes during sickness in more detail [20].

The aim of this study was to investigate and deepen the knowledge about the various motivational aspects of sickness behavior, using an experimental inflammatory model induced by lipopolysaccharide (LPS) [[21], [22]] in a within-subject, placebo-controlled designed. Immune responses are metabolically costly, requiring increased energy for immune cell activity and the maintenance of elevated core temperature [23]. From an allostatic perspective [24], this increased demand necessitates a reorganization of motivational priorities aimed at conserving energy and maintaining internal stability. Behaviors such as *reduced physical activity* and *increased rest and sleep* would support internal regulation by minimizing energy expenditure and reallocating resources toward immune defense and tissue repair. Simultaneously, *decreased motivation for social activities* may limit exposure to social stressors or risk for secondary infections, while also reducing the cognitive and energetic costs of social engagement. *Increased motivation to seek care* would serve as a complementary strategy to mobilize external support systems (e.g. medical treatment, comfort, protection) to compensate for the heightened allostatic load and to favor the return to homeostasis. Furthermore, *reduced hunger* would reflect a temporary suppression of energy intake to minimize digestive burden and pathogen exposure, whereas *thirst* may be maintained or increased to support hydration and thermoregulation; while *food preference* would shift away from rich or hard-to-digest foods and toward lighter or hydrating options. Such appetite and preference changes may serve a defensive function, or “starving the pathogen” by limiting access to nutrients, in particular iron, needed for microbial growth [25]. Furthermore, a transient motivational reallocation would occur during sickness, involving a shift in the valuation of expected rewards and in the appraisal of effort costs [26,27]. Based on reinforcement learning framework [28,29], such shift would shape motivational behavior. Hence, the perceived cost of energy-intensive behaviors such as *physical activity* and *social activities* would increase during sickness, while the reward value of low-effort recovery-supportive behaviors like *rest and sleep* and *care seeking* would become more salient. This increase in value would also be translated into an *increased willingness to use (monetary) resources* in order to be able to e.g. sleep.

We hypothesized that LPS-induced sickness would be associated with a rearrangement of motivational goals reflecting energy preservation, including increased motivation to rest, to sleep, to be alone, and to be at home. Additionally, we hypothesized that motivation favoring recovery, such as being taken care of by a partner, parent, or at a hospital, would increase during experimentally induced sickness. We also explored the association between the self-rated motivation in different motivational categories and an overall self-rated measurement of sickness behavior, concentration of inflammatory markers, and body temperature at the peak of the sickness response.

## 2 Methods

### 2.1 Participants

Twenty-two healthy volunteers were recruited via flyers at university campuses in Stockholm. Before inclusion, participants underwent a complete medical examination, including blood samples and electrocardiogram. Exclusion criteria included any physiological and psychiatric disease, medication, high body mass index, smoking, and high alcohol consumption (see Ref. [12] for detailed list of exclusion criteria). One participant was excluded because of elevated baseline concentrations of interleukin (IL)-6 (value > 4 standard deviations from mean) and tumor necrosis factor-alpha (TNF-α) (value > 3 standard deviations from mean). The sample size was consequently reduced to twenty-one (9 women) aged 19 to 34 (*M* = 23, *SD* = 4).

### 2.2 Study design

This study derives from a data collection conducted in 2015 [12] (preregistered on ClinicalTrials.gov: NCT02529592), which used a balanced within-subject placebo-controlled crossover design with a three-to-four-week washout period, and a double-blind study design. Participants arrived at the laboratory between 07:30–08:00 a.m. and the injection of LPS/placebo was made between 08:30–09:00 a.m. Participants were instructed to only eat a light breakfast before arriving and were offered a light lunch at 12:30 p.m. (i.e. ∼4h post-injection). Several behavioral tasks such as the ‘Effort Expenditure for Reward Task’ [12] were made 1–6 h after injection. All participants participated on two occasions, one in which they received an intravenous injection of LPS at a dose of 2.0 ng/kg of body weight, and one in which they were injected with a placebo (NaCl 0.9 %). Only the responsible physician was aware of whether LPS or placebo was injected. The study was approved by the regional ethical review board in Stockholm, Sweden (2014/1946–31/1, 2015/1415–32, 2021-05835-02). Participation was compensated with 3500 SEK.

### 2.3 Measurements

#### 2.3.1 Self-rated motivation during sickness

To measure self-rated motivation to engage in various behaviors, the Motivation Scale of Sickness (MOSSick) was administered at baseline (before injection), at the peak of the sickness response (3 h post-injection), and at the end of the sickness response (7.5 h post-injection). MOSSick is a modified version of the ‘Motivation Scale of Sleepiness’ (MOSS), which previously has been used in the context of sleep restriction [30]. MOSSick contains 26 items aimed at measuring motivation to engage in behaviors, which we grouped in seven categories for easing of interpretation: ‘hunger and thirst’, ‘food preferences’, ‘physical activities’, ‘rest and sleep’, ‘social activities’, ‘care seeking’, and ‘utilization of resources’ (i.e., willingness to ‘pay’ and ‘walk’ to be able to rest or to be healthy right away). The willingness to pay is taken from behavioral economics [31], to measure the subjective valuation of resting and recover. The willingness to walk measures the balance between increased cost of physical effort and increased value of resting/recovering. The MOSSick items are described in details in Table 1. Compared to the MOSS, the MOSSick includes three items specific to the state of sickness (‘How much would you be willing to pay to become healthy right away?‘, ‘How much would you like to be healthy right now?‘, ‘How much would you like to receive care at a hospital right now?‘). All items were presented in Swedish. All items, apart from the ones in the ‘utilization of resources’ category, were answered on a five-point Likert-type scale, with answers ranging from ‘not at all’ to ‘very much’. Items ‘pay’ and ‘walk’ in the category ‘utilization of resources’ were answered on a scale with either: 0 SEK, 20 SEK, 50 SEK, 100 SEK, and 500 SEK, or 0 km, 1 km, 2 km, 4 km, 8 km, and 12 km as response alternatives, along with an alternative to input any amount or distance freely; or if they did not want to choose one of alternatives, they could check a box labelled: ‘not applicable’.

**Table 1:** Effect of LPS vs placebo administration on the items of the ‘Motivation Scale of Sickness’ (MOSSick).

Category	Item number	Question	Effect of Time (vs baseline, in placebo)				LPS vs Placebo at baseline		Effect of LPS (vs placebo) ∗ time			
			3 h		7.5 h		*b (SE)*	*p*	3 h		7.5 h	
			*b (SE)*	*p*	*b (SE)*	*p*			*b (SE)*	*p*	*b (SE)*	*p*
Hunger & thirst	1.	How hungry are you?	**2.84 (0.33)**	**<0.001**	−0.07 (0.43)	0.86	−0.19 (0.37)	0.61	**−0.90 (0.44)**	**0.04**	−0.11 (0.44)	0.80
	12.	How much would you like to drink a glass of water right now?	−0.25 (0.49)	0.60	−0.21 (0.48)	0.66	−0.14 (0.39)	0.71	**1.48 (0.63)**	**0.02**	0.34 (0.59)	0.57
	14.	How much would you like to have a meal right now?	**2.81 (0.42)**	**<0.001**	0.20 (0.46)	0.66	−0.12 (0.32)	0.69	**−1.58 (0.58)**	**0.006**	−0.22 (0.51)	0.67
Food preferences	15.	How much would you like to eat a fruit right now?	**1.98 (0.49)**	**<0.001**	0.57 (0.35)	0.10	**−0.88 (0.39)**	**0.03**	−0.35 (0.66)	0.59	0.13 (0.61)	0.83
	16.	How much would you like to eat candy (or something else that is sweet) right now?	**1.65 (0.43)**	**<0.001**	**0.95 (0.49)**	**0.05#**	**−0.73 (0.38)**	**0.05#**	0.12 (0.58)	0.83	1.13 (0.60)	0.06
	17.	How much would you like to eat a juicy steak (or something else that is protein-rich) right now?	**1.27 (0.33)**	**<0.001**	0.14 (0.17)	0.42	−0.40 (0.27)	0.15	−0.80 (0.44)	0.07	−0.29 (0.41)	0.48
Physical activities	5.	How much would you like to go for a walk right now?	−0.07 (0.44)	0.87	0.27 (0.48)	0.57	−0.56 (0.47)	0.23	**−2.06 (0.79)**	**0.009**	−0.21 (0.64)	0.74
	6.	How much would you like to exercise right now?	0.39 (0.30)	0.20	0.61 (0.33)	0.06	0.16 (0.45)	0.72	**−4.17 (1.21)**	**<0.001**	**−2.65 (0.77)**	**<0.001**
	13.	How much would you like to go shopping for food right now?	**1.51 (0.40)**	**<0.001**	**1.61 (0.42)**	**<0.001**	−0.41 (0.33)	0.21	**−1.99 (0.53)**	**<0.001**	**−1.92 (0.59)**	**0.001**
Rest and sleep	2.	How much would you like to rest right now?	0.19 (0.39)	0.64	−0.26 (0.51)	0.62	0.16 (0.38)	0.67	**2.00 (0.75)**	**0.008**	0.46 (0.66)	0.48
	3.	How much would you like to lie down in your own bed (and sleep) right now?	−0.57 (0.34)	0.09	−0.72 (0.53)	0.17	−0.30 (0.44)	0.49	**2.93 (0.63)**	**<0.001**	**1.41 (0.70)**	**0.04**
	4.	How much would you like to lie down in the closest available bed (and sleep) right now?	−0.08 (0.41)	0.85	**−1.13 (0.43)**	**0.008**	0.17 (0.42)	0.68	**1.65 (0.61)**	**0.007**	0.31 (0.61)	0.61
	7.	How much would you like to be at home right now?	0.65 (0.39)	0.10	**1.00 (0.49)**	**0.04**	0.47 (0.46)	0.30	**1.50 (0.48)**	**0.002**	0.83 (0.56)	0.14
Social activities	8.	How much would you like to be alone right now?	−0.16 (0.38)	0.67	0.69 (0.47)	0.14	−0.54 (0.47)	0.25	**2.85 (0.53)**	**<0.001**	0.13 (0.60)	0.83
	9.	How much would you like to be with friends right now?	−0.02 (0.35)	0.95	0.39 (0.39)	0.32	−0.49 (0.45)	0.27	**−2.18 (0.72)**	**0.002**	−0.60 (0.60)	0.31
	10.	How much would you like to socialize with a stranger right now?	**−0.81 (0.38)**	**0.04**	−0.98 (0.55)	0.08	−0.84 (0.59)	0.15	−0.78 (0.71)	0.27	−0.31 (0.72)	0.67
	11.	How much would you like to go on a date with a stranger right now?	0.11 (0.34)	0.75	0.23 (0.35)	0.51	−0.38 (0.43)	0.37	**−2.64 (1.04)**	**0.01**	**−1.70 (0.84)**	**0.04**
Care seeking	18.	How much would you like to be healthy right now?	−0.40 (0.30)	0.19	0.23 (0.47)	0.63	0.00 (0.39)	1.00	0.49 (0.57)	0.39	−0.35 (0.72)	0.63
	19.	How much would you like to receive care by your partner right now?	−0.08 (0.30)	0.80	−0.57 (0.37)	0.13	−0.08 (0.38)	0.84	**1.33 (0.44)**	**0.003**	**1.20 (0.52)**	**0.02**
	20.	How much would you like to receive care by your mother (father) right now?	−0.41 (0.44)	0.34	**−1.02 (0.45)**	**0.02**	−0.74 (0.43)	0.09	**2.52 (0.64)**	**<0.001**	**1.59 (0.62)**	**0.01**
	21.	How much would you like to receive care at a hospital right now?	−0.29 (0.41)	0.48	**−1.33 (0.54)**	**0.01**	0.04 (0.43)	0.93	**2.04 (0.58)**	**<0.001**	0.64 (0.73)	0.38
	22.	How much would you like to receive a hug from someone at work or that you know casually right now?	−0.34 (0.33)	0.31	**−0.82 (0.41)**	**0.05**	**−1.12 (0.48)**	**0.02**	1.24 (0.65)	0.06	**1.33 (0.65)**	**0.04**
Utilization of resources (Pay and Walk)	23.	How much would you be willing to pay to go to bed right now?	−0.16 (0.51)	0.76	−0.62 (0.73)	0.40	−1.09 (0.77)	0.15	**3.71 (1.00)**	**<0.001**	1.91 (1.16)	0.10
	25.	How much would you be willing to pay to become healthy right away?	0.05 (0.26)	0.83	−1.88 (1.30)	0.15	−0.27 (1.32)	0.84	**2.45 (1.01)**	**0.02**	2.00 (1.68)	0.23

Notes: The MOSSick categories were defined in accordance with the theoretical framework, with items grouped into domains that capture conceptually related aspects of motivational drives during sickness, and should not be interpreted as validated dimensions. Generalized estimating equations (GEE's) using cumulative logit marginal models for each MOSSick item in the lipopolysaccharide (LPS) and the placebo condition, at baseline, after 3 h, and 7.5 h post-injection. MOSSick items were answered using a 5-point Likert scale (from “not at all” to “very much”, except items 23 and 25 that are answered in Swedish crowns and transformed into categories (0–5 SEK = 1, 20 SEK = 2, 50 SEK = 3, 100 SEK = 4, and 500 SEK = 5). Analyses were not conducted for items 24 and 26 (answered in km) because of too many missing or “not applicable” values. A time exchangeable local odds ratio structure was used, except for item 22 where a uniform local odds ratio structure was used because the model did not converge. Estimated coefficient (b), Standard error (SE). # = statistical trend. P-values were not corrected for multiple comparisons because we conducted analyses of conceptually distinct aspects of motivational drives captured by the individual MOSSick items based on *a priori* hypotheses.

#### 2.3.2 Measures of sickness

The Sickness Questionnaire; SicknessQ [32] was used to assess sickness behavior. The scale contains 10 items, rated on a Likert-type 4-point scale, ranging from ‘strongly disagree’ to ‘strongly agree’. Items include: ‘I want to keep still’, ‘My body feels sore’, ‘I wish to be alone’, ‘I don't wish to do anything at all’, ‘I feel drained’, ‘I feel nauseous’, ‘I feel shaky’, ‘I feel tired’, ‘I feel depressed’, and ‘I have a headache’. The questionnaire was administered before the injection and 1.5 h, 3 h, 5 h, and 7.5 h post-injection.

#### 2.3.3 Inflammatory markers

To measure blood concentration of IL-6, IL-8, and TNF-α, blood samples were drawn before and 1 h, 1.5 h, 2 h, 3 h, 4 h, 5 h, and 7 h post-injection. Plasma cytokine concentrations were analyzed using high-intensity multiplex (Human Mag Luminex Performance Assay, LHSCM000, LHSCM206, LHSC208, and LHSC210, RnD Systems, MN, USA). IL-6 and TNF-α concentrations were used in the current study as indices of the inflammatory response.

#### 2.3.4 Body temperature

Tympanic temperature was measured before injection and subsequently every 30 min up until 7.5 h post-injection.

### 2.4 Statistics

Statistical analyses were conducted in R [33], the code can be found in the Supplementary text.

Given that our aim was to estimate population-level effects of inflammation on symptom ratings, we used population-averaged ordinal generalized estimating equations (GEE), allowing explicit modeling of the within-participant correlation structure. GEE were conducted using the R package ‘multgee’ with the ‘ordLORgee’ function [34]. This model, adapted for ordinal and longitudinal data, was used to assess the effects of condition (LPS, vs placebo), time (3 h and 7.5 h vs baseline, in the placebo condition), and the effect of time in the LPS condition compared to the placebo condition, on self-rated motivation (MOSSick items, score 1–5). A time exchangeable local odd ratio structure was applied, which assumes that the local odds ratios are the same across different time points for a given pair of categories, and study day and time were entered as repeated variables; with the following R code: ordLORgee (MOSSick_item ∼ factor (Condition [placebo = 0; LPS = 1])∗factor (Time [baseline = 0; 3 h = 1; 7.5 h = 2]), id = id, repeated = interaction (Day,Time), link = "logit", LORstr = "time.exch", data = df). Items 23 (pay to go to bed) and 25 (pay to be healthy) were transformed into categories (1–5 to follow similar score as the other items) before analyses (0Kr or 5Kr = 1, 20kr = 2, 50Kr = 3, 100Kr = 4, 500Kr = 5). For item 22 (receive a casual hug), the time exchangeability local odds ratio structure did not converge, and a uniform local odds ratio structure was used. Item 24 (walk to go to bed) and item 26 (walk to be healthy) were excluded from the analyses because too many participants answered ‘not-applicable’ (34.9 % and 54.8 % respectively). The remaining MOSSick ratings as well as the SicknessQ ratings contained no missing data.

GEE is a regression framework, it models the log-odds of the ordinal outcome as a function of predictors, yielding population-averaged effects accounting for within-subject correlations. In this framework, with placebo/baseline as reference levels:

• the Condition coefficient contrasts LPS vs placebo when Time is at the reference level, i.e. *at baseline.* It answers: at baseline, how do ratings differ between LPS and placebo?
• the Time coefficients contrast 3 h and 7.5 h vs baseline, when Condition is at the reference level, i.e. *in the placebo group.* It answers: in the placebo condition, how do ratings at 3 h and 7.5 h differ from baseline?
• and the interaction terms indicate how these time contrasts change in LPS relative to the time contrasts in placebo. It answers: is the change from baseline (baseline to 3 h and baseline to 7.5 h) differ after LPS compared to after placebo? Interaction terms can be interpreted as follows:
  o If the main effect of Time (in placebo) is *not* significant (meaning there is not change between 3 h/7.5h and baseline in the placebo condition), and the interaction term is significant, this indicates a significant change from baseline in the LPS condition.
  o If the main effect of Time (in placebo) is significant (meaning there is a significant change between 3 h/7.5h and baseline in the placebo condition), and the interaction term is *not* significant, then this means that the change over time in the LPS condition is similar to that observed in the placebo condition.
  o If both the main effect of time in the placebo condition *and* the interaction LPS ∗ time are significant, then this means that the change from baseline in the LPS condition differs from the change from baseline in the placebo condition. In this case, post-hoc GEEs using time as factor in the LPS condition only are needed to verify if there is a significant change from baseline in the LPS condition.

Although twenty-four GEE analyses were conducted, we did not conduct any Bonferroni correction. The reason is that the analyses are not repeated tests of a single hypothesis, but rather separate analyses of conceptually distinct aspects of motivational drives captured by the individual MOSSick items based on *a priori* hypotheses. In such cases, correction for multiplicity is not generally recommended, as it can inflate type II error and obscure meaningful effects [35,36].

In order to explore the association between MOSSick item categories and the magnitude of the sickness response measured via SicknessQ score, tympanic temperature, IL-6 concentrations, and TNF-α concentrations, correlations were conducted using the data at 3 h post-injection, corresponding to the peak of sickness behavior measured with the SicknessQ, and plateau of the fever response [12]. The MOSSick categories were defined in accordance with the theoretical framework, with items grouped into domains that capture conceptually related aspects of motivational drives during sickness. Each item score was delta-transformed (LPS data 3 h post-injection minus placebo data 3 h post-injection). MOSSick item 8 (be alone) was reversed to match expected direction in its category (i.e., higher ratings as positive). Delta-scores of the MOSSick items in each MOSSick category were then summed up and correlated with the sickness measures using Kendall's tau-b through the base R function ‘cor.test’ [33]. Kendall's tau-b is used to assess monotonic relationships in ordinal or continuous data, as it is generally more robust to outliers than spearman's rho [37]. Sensitivity analyses of the correlations were conducted, removing items that assess motivational aspects of sickness behavior in the SicknessQ (i.e. removing ‘I want to be still’, ‘I wish to be alone’, and ‘I don't wish to do anything at all’), as well as removing one outlier in cytokine values.

## 3 Results

### 3.1 Motivational changes during experimentally induced sickness

**Fig. 1:**
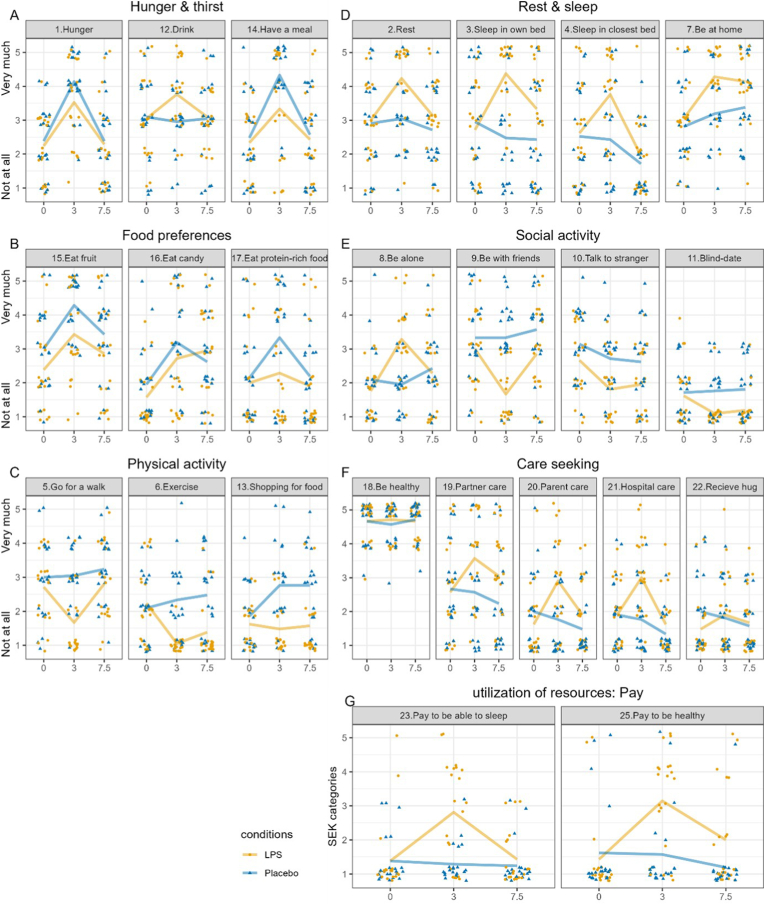
Illustration of the mean motivation to engage in behaviors measured with the MOSSick, in response to LPS and placebo administration. Items are ordered by category of the MOSSick. The MOSSick categories were defined in accordance with the theoretical framework, with items grouped into domains that capture conceptually related aspects of motivational drives during sickness, and should not be interpreted as validated dimensions. Numbers in title in grey areas indicate item number, item 24 and 26 were excluded from the analyses and figure (See full list with item names in Table 1). Jittered dots indicate individual ratings in the placebo condition (blue triangles) and the LPS condition (orange dots) on each MOSSick item at baseline and at 3 and 7.5 h post injection. Lines indicate mean ratings in the placebo condition (blue line) and the LPS condition (orange line) at each timepoint. MOSSick items are answered using a 5-point Likert scale (from “not at all” to “very much”, except items 23 and 25 that are answered in Swedish crowns and transformed into categories (0–5 SEK = 1, 20 SEK = 2, 50 SEK = 3, 100 SEK = 4, and 500 SEK = 5).

MOSSick ratings over the day in the placebo and the LPS conditions are presented in Table 1, Fig. 1, and summarized below.

#### 3.1.1 Hunger and thirst

Participants were more hungry and more motivated to have a meal 3 h post-placebo injection compared to baseline (main effect of Time), although this effect was larger in the placebo condition as compared to the LPS condition (negative interaction effect). Post-hoc tests revealed that participants still reported being hungrier (B(SE) = 1.52 (0.38), p < .001) and more motivated to have a meal (B(SE) = 1.40 (0.43), p = .001) 3 h post-LPS injection compared to baseline (Fig. 1A). Participants also reported being thirstier 3 h post-LPS injection compared to placebo (positive interaction effect, Fig. 1A).

#### 3.1.2 Food preference

Three hours post-placebo injection, participants were more willing to eat a fruit, sweets, and protein-rich food compared to before the injection (main effect of Time). The motivation to eat sweets was also increased at 7.5 h (p = .05) in the placebo condition (main effect of Time). The ratings for eating a fruit and sweets were lower at baseline in the LPS condition compared to the placebo condition (main effect of Condition), but the increase in the willingness to eat a fruit, sweets, and protein-rich food from baseline to post-injection were similar between the LPS and placebo conditions (no significant interaction) (Fig. 1B).

#### 3.1.3 Physical activity

Participants in the placebo condition reported being more motivated to go shopping for food at both 3 and 7.5 h post-injection compared to baseline (main effect of Time). This effect of time was significantly lower in the LPS condition (negative interaction effect), and post-hoc analyses indicated that there was no significant change in the motivation to go shopping for food throughout the day after the LPS injection (3 h: B(SE) = -0.74 (0.54), p = .17; 7.5 h: B(SE) = -0.57 (0.49), p = .24) (Fig. 1C). Participants showed reduced motivation to go for a walk and to exercise 3 h post-LPS injection compared to placebo (negative interaction effect), and motivation to exercise was also reduced 7.5 h post-LPS injection (negative interaction effect) (Fig. 1C).

#### 3.1.4 Rest and sleep

In the placebo condition, participants reported being less motivated to lie down and sleep in any bed, and more motivated to be at home 7.5 h post-injection (main effect of Time). In comparison, the motivation to rest, to lie down and sleep in their own bed or in any bed, and to be at home was increased 3 h post LPS injection compared to placebo injection (positive interaction effect), and motivation to lie in their own bed was still higher 7.5 h post-LPS injection (positive interaction effect) (Fig. 1D).

#### 3.1.5 Social activity

Participants reported less motivation to socialize with a stranger 3 h post-placebo injection compared to baseline (main effect of Time), and this effect was similar in the LPS condition (no interaction effect). In the LPS condition, participants reported increased motivation to be alone (positive interaction effect), and lower motivation to be with friends and to go on a date with a stranger 3 h post-injection compared to placebo (negative interaction effect) (Fig. 1E). The motivation to go on a date was also lower at 7.5 h post-LPS injection compared to placebo (negative interaction effect) (Fig. 1E).

#### 3.1.6 Care seeking

In the placebo condition, the willingness to receive care by a parent or at a hospital, as well as to receive a casual hug, decreased 7.5 h after injection compared to baseline (main effect of Time). The injection with LPS instead led to an increased willingness to seek care by a parent or at a hospital 3 h post-injection compared to placebo (positive interaction effect). The motivation to receive a hug was lower at baseline in the LPS condition compared to the placebo condition (main effect of Condition). The motivation to receive care by a parent or to receive a casual hug was higher 7.5 h post-LPS injection than in the placebo condition (in which the motivation decreased at that time point) (positive interaction effect), and post-hoc analyses revealed no significant change from baseline (post-hoc baseline vs 7.5 h in LPS condition: care by a parent: B(SE) = 0.69 (0.37), p = .06; casual hug: B(SE) = 0.49 (0.42), p = .25) (Fig. 1F). The injection with LPS also led to an increased willingness to seek care by a partner 3 h and 7.5 h post-injection compared to placebo (positive interaction effect; Fig. 1F). Self-rated willingness to be healthy was not altered post-injection in the placebo or in the LPS condition (no significant effect; Fig. 1F).

#### 3.1.7 Utilization of resources

There were no statistically significant changes across time in the placebo condition in the willingness to pay in order to sleep or be healthy (no main effect of Time). In the LPS condition, participants were willing to pay more to be healthy at once and to go to bed, 3 h post-LPS injection compared to placebo (positive interaction effect; Fig. 1G).

### 3.2 Associations between motivational changes during sickness and intensity of the sickness response 3 h post-LPS injection

**Fig. 2:**
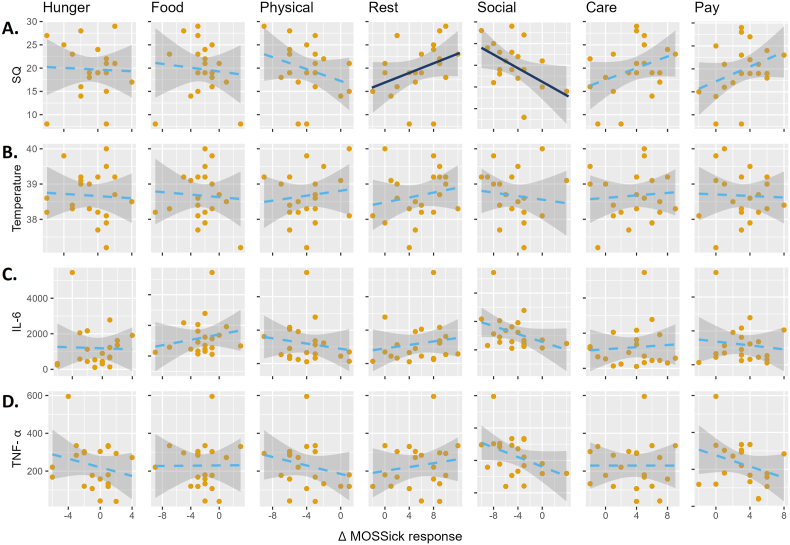
Associations between sickness measures and the LPS-induced changes in motivation to engage in behaviors measured with the MOSSick at 3 h post LPS-injection. The MOSSick categories were defined in accordance with the theoretical framework, with items grouped into domains that capture conceptually related aspects of motivational drives during sickness, and should not be interpreted as validated dimensions. A: sickness behavior measured with Sickness Questionnaire (SicknessQ, score = 0–30); B: tympanic temperature (°C); C: IL-6 plasma concentrations (pg/mL); D: TNF-α concentrations (pg/mL); during the peak of the sickness response (3 h post-injection) in the LPS condition. X-axes indicates delta scores (LPS-placebo at 3 h post-injection) in each MOSSick-category. Dark-blue solid lines indicate significant associations (p < .05) and light-blue dashed lines indicates non-significant associations. Abbreviations: MOSSick: Motivation scale of sickness, Temperature: tympanic temperature, SQ: Sickness questionnaire, LPS: Lipopolysaccharide, IL6: Interleukin-6, TNF-α: Tumor necrosis factor-alpha.

Stronger sickness behavior (measured with the SicknessQ*)* 3 h post-LPS injection was associated with higher MOSSick delta scores in the category ‘rest and sleep’ [τb = 0.35, p = .04], and with lower scores in the category ‘social activities’ [τb = −0.36, p = .03] (Fig. 2A, Supplementary Table S1). This indicates that participants who reported stronger sickness behavior reported a higher motivation to rest/sleep and a lower motivation for social activities. When removing the items that measured motivational aspects of sickness behavior in the SicknessQ, only the association with social activities remained [τb = -0.33, p = .048] **(**Supplementary Table S1).

No other significant correlations were found (see Fig. 2 and Supplementary Table S1; sensitivity analyses without the outlier in IL-6 and TNF-α concentrations are presented in Supplementary Table S2).

## 4 Discussion

The current study offers an initial characterization of the changes in subjective motivational drives occurring in humans during sickness. Eighteen out of 24 self-assessed motivational aspects were altered during experimental sickness induced by administration of LPS compared to placebo. As hypothesized, sickness was associated with reduced motivation for physical activities, social withdrawal, and increased motivation for activities favoring energy preservation, such as resting, lying down and sleeping in a bed, and being at home; and to obtain care by a partner, parent, or at the hospital. Interestingly, many of these aspects remained significantly elevated 7.5 h post-LPS injection, i.e. when most of the other sickness symptoms have subsided (but when fatigue can remain elevated). Participants were also willing to pay more in order to go to sleep and to be healthy at once as an effect of experimentally-induced inflammation. Overall appetite was reduced, but not completely abolished, during sickness compared to placebo, and food preferences were not strongly affected. During the peak of the sickness response, motivation to rest and to avoid social activities was associated with overall ratings of sickness behavior (total SicknessQ scores), but the other measures of the inflammatory response (fever, cytokine concentrations) were not associated with the inflammation-induced changes in the motivational ratings – although sample size was limited for such analyses.

The motivational changes observed during sickness are in line with the notion that sickness behavior favors energy preservation and recovery [1,2,6]. Our current findings also highlight the complexity of the social aspects of sickness behavior; sick participants wanted to withdraw socially with respect to friends and random strangers, but displayed a willingness to approach others who would provide care. This notion concurs with points made by us and other authors [38,39]. Inflammation indeed alters threat- and reward-related neural sensitivity, resulting in reduced motivation to partake in risky social experiences, but also in increased motivation to partake in supportive social experiences [39,40]. As Muscatell and Inagaki [41] point out, social withdrawal or approach is related to closeness to the other involved person(s). Motivation for other social activities may therefore be reduced partly because of being more distant to the persons involved. However, in the current study, motivation to seek care at a hospital (i.e., by strangers) increased in the sick participants, indicating that, during sickness, the motivation to recover through care counteracts the drive for social isolation, or that our need to be cared for and ultimately our most basic need to recover is the underlying motivator. These notions are supported by a recent study, in which we show that sick participants express increased motivation to obtain care from individuals who can provide care and support, i.e., caregivers, not just healthcare professionals but also other strangers [38]. Importantly, the increased self-rated motivation to seek care by unknown caregivers during sickness implies that the idea that sickness makes us less social is too general. Rather, it showcases functional flexibility and context dependence. Accordingly, describing sickness behavior as a motivational reorganization favoring energy preservation, caring, and recovery is more accurate [5,20,41,42]. These findings highlight the complexities of social motivational changes occurring during the inflammatory response, and speaks for a need for a higher resolution in the discussion about immune-defense mechanisms.

Interestingly, the current study found that overall appetite was reduced but not abolished in sick participants. Increase in appetite was observed also in the LPS condition (although to a lesser extent than in the placebo condition), and no substantial shifts in food preferences occurred. Loss of appetite is often described as a part of the sickness response [2,8] and has been hypothesized to occur in order to ‘starve the pathogen’ [25]. However, reducing food consumption altogether would also reduce energy available for immune functions and heat-generative mechanisms [5,43]. Participants in the current study reported an overall increased appetite, as well as increased preferences for sweets, fruits, and protein-rich food, after the injection both when healthy and when sick. This effect is likely due to the fact that participants arrived early in the morning having eaten a light breakfast, and lunchtime was quite late during the day, 4–5 h post-injection. Therefore, the symptom would more accurately be described as “reduced appetite” rather than a complete “loss of appetite”.

The MOSSick has been derived from the MOSS, Motivation Scale of Sleepiness, which was developed to assess motivational drives related to sleepiness and how we organize behavior to assure sufficient sleep [30]. Several motivational alterations observed during sickness in our study were very similar to those reported after sleep loss. Sleep deprivation increased the motivation to rest, sleep, and seek care by a partner, but reduced motivation to engage in physical and social activities [30]. This similarity is not surprising, given that motivational changes during sickness and after sleep deprivation should both support the needs for energy preservation, care, and recovery. Furthermore, inflammation also leads to an increase in sleepiness [44]. Contrary to sickness, however, sleep deprivation did not induce an increased willingness to obtain care from a parent, and was associated with no strong changes in appetite. Using the same scale in our recent “two-hit” study, in which we assessed the effect of sleep deprivation and COVID-19 vaccination alone and in combination [19], we showed consistent effects: sleep deprivation and vaccination-induced low-grade inflammation were both associated with sleep preparatory behaviors and reduced motivation for physical and social activities (with some additive effects but no strong evidence of an interaction of the two hits). In that study, appetite was not affected by vaccination – most likely because of the low level of inflammation. Together with the present data, this suggests that the motivational drives related to energy preservation during sickness might be particularly associated with sleepiness/fatigue, while other motivational changes such as appetite and desire for care could be specific to the severity and type of sickness. Although we did not observe any significant correlations between the intensity of the sickness responses and changes in appetite and desire for care in our study, this may be due to restricted variability in sickness severity in our sample (with most participants showing relatively strong responses) or to non-linear relationships in which effects on these symptoms only emerge above certain severity thresholds.

Although the current findings provide an insight into the motivational changes occurring during acute sickness, we need to keep in mind that motivations are highly dependent on context and other external demands. As showcased in Aubert et al [7,8], the behavioral changes occurring during sickness are dependent on the need to find or provide safety for oneself or one's offspring. In the current study, the increased motivation to rest and the reduced motivation to move and socialize could partly be explained by the fact that the participants had no personal duties to take care of during the study day, and possible expectations that their needs for care would be satisfied, making motivation to rest and to reduce activity possible. In future studies, it would be interesting to modulate environmental factors to investigate how these would affect subjective motivation during sickness. Furthermore, subjective motivational states are not always reflected in objective behavioral changes [12], and incorporating both self-reports and objective behavioral changes of goal-directed effort would provide a more comprehensive overview of the motivational changes during sickness.

An unexpected result was the absence of any significant association between the measures of the inflammatory response and the motivational drives. This finding should be interpreted with caution given the small sample size, but could indicate a ceiling effect, an “on/off” effect, or a distinction between the peripheral vs central sickness responses. The symptoms induced by the dose of LPS at 2.0 ng/kg body weight are quite intense in a large proportion of participants, and might therefore be compressed in the extreme category ranges. In our recent study with vaccination [19], concentrations of cytokines at low-grade levels were correlated with motivational changes, supporting this possibility. Alternatively, it could be that once the production of cytokines reaches a certain level, changes in motivational drives are triggered in a non-linear way and irrespective of the cytokine concentrations. Our previous study suggests that the subjective sickness responses might not always be related to the intensity of the peripheral immune responses, possibly because of the involvement of top-down factors such as individual's expectations [45] or factors such as central sensitivity [46]. Considering the low sample size, future studies will be needed to address this issue, for instance by including intermediate inflammatory challenges and additional biological markers (e.g. additional cytokines and neuroendocrine hormones) and neuroimaging correlates.

There are a number of limitations to consider. Firstly, there were a few items for which differences were observed between the two conditions at baseline. Because of the within-subject design effect, participants might have been in a different motivational state at baseline for various reasons. Furthermore, although self-assessment is a good way of tapping into a person's experienced motivational drives, this way of measuring also has some downsides. For example, only motivation that the individual is aware of can be assessed, and there is a risk that the participants misunderstand or do not answer truthfully because of e.g., difficult questions, fatigue, social desirability, and self-serving bias [47,48]. Nevertheless, self-assessed motivation during sickness is needed, given that the subjective motivational state is likely to drive observational behavioral changes. Another limitation is that the sample contained healthy young individuals in Sweden, mostly students, limiting the possibility for generalizations to the larger population. For example, motivational changes may differ depending on mental health [49,50], culture [17], and perceived social status [51]. Again, the sample size and power were limited, particularly for making inferences about small-to mid-sized effect sizes. However, since the immune and behavioral effects effect of LPS are very strong, this risk is partly attenuated, at least for the main effects of LPS [[12], [22]], although the absence of correlations may reflect type II errors. To widen the knowledge of motivation during sickness, we made separate analyses for all 24 items separately, which, together with the limited sample size, increased the risk for inflated effect sizes and type-I errors. To reduce the number of correlational analyses, theses were conducted by category, resulting in 28 correlational analyses. Nevertheless, they were conducted on sickness measures from only the LPS condition at 3 h post-injection, resulting in much fewer datapoints than ideal, increasing the risk of both type-I and type-II errors. The pattern of the results was logic and largely expected, but further studies are needed to test the robustness, as well as how factors such as gender, age, and how naturally occurring sickness influence motivational drives during sickness.

Importantly, the MOSSick has not yet been validated as a measure of motivation during sickness, and we recommend psychometric evaluations in the future. In particular, the validity and understanding of the items “willingness to walk” – for which a large number of participants answered “not applicable” – should be assessed. Furthermore, we decided to transform the answers on the “willingness to pay” items, because they are discrete, ordered values and thus not continuous, and to harmonize the scoring with the other MOSSick items, which are ordinal. However, this could also hide the true economic distances between values, and thus should be addressed in future studies. Finally, we grouped items in dimensions for the correlation analyses based on the theoretical framework, and not on psychometric analyses. We observed different effects between items from the same theoretical dimensions, indicating that the dimensions should not be interpreted as validated dimensions and that future studies should conduct dimension reduction analyses.

To conclude, our findings suggest that motivational changes in sick individuals favor energy preservation and recovery, in line with the adaptive theory of sickness behavior [2,5]. Furthermore, the social aspects of sickness highlight the need for care, with increased motivation to receive care by close others as well as by strangers [38]. Appetite was reduced, but not completely abolished, during sickness. Sick participants were willing to pay more to sleep and to be healthy at once. Altogether, our findings illustrate the complexity of sickness-related motivational drives, which are centered around providing the necessary means for the infected individuals to cope with, and recover from illness. However, these results should be interpreted with caution, as they are based on a small, young, and healthy sample, which limits the generalizability of the conclusions. The MOSSick may serve as a useful tool in future studies, given that no validated scale currently exists to assess motivational dimensions. However, a validation study and additional research in more diverse samples will be needed to further validate its utility in research and clinical studies.

## CRediT authorship contribution statement

**Rasmus Skarp:** Writing – original draft, Visualization, Formal analysis, Data curation. **Lina S. Hansson:** Writing – review & editing, Visualization, Supervision, Formal analysis. **Tina Sundelin:** Writing – review & editing, Supervision, Methodology. **Sofie Paues:** Writing – review & editing, Methodology, Investigation. **Martin Janson:** Writing – review & editing, Visualization, Formal analysis, Data curation. **Leonie JT. Balter:** Writing – review & editing, Methodology, Formal analysis. **Mats J. Olsson:** Writing – review & editing, Methodology, Funding acquisition, Conceptualization. **John Axelsson:** Writing – review & editing, Supervision, Methodology, Conceptualization. **Mats Lekander:** Writing – review & editing, Supervision, Resources, Project administration, Funding acquisition, Conceptualization. **Julie Lasselin:** Writing – review & editing, Supervision, Resources, Project administration, Methodology, Investigation, Funding acquisition, Formal analysis, Data curation, Conceptualization.

## Funding

This work was supported by the Swedish Foundation for Humanities and Social Sciences [grant number P12-1017 to MJO] and the 10.13039/501100004359Swedish Research Council [grant number 421-2012-1125 to MJO; 2020-01606 to JL].

## Declaration of competing interest

The authors declare the following financial interests/personal relationships which may be considered as potential competing interests: Julie Lasselin currently serves as Associate Editor of *Comprehensive Psychoneuroendocrinology* and has been appointed Editor-in-Chief effective January 2026.

Julie Lasselin reports financial support was provided by Swedish Research Council. Mats J Olsson reports financial support was provided by Bank of Sweden Tercentenary Foundation. Mats J Olsson reports financial support was provided by Swedish Research Council.

If there are other authors, they declare that they have no known competing financial interests or personal relationships that could have appeared to influence the work reported in this paper.
