# Supplementary material for: The motivational drives of sickness: Acute changes in self-rated motivation during experimental endotoxemia assessed with the newly developed Motivation Scale of Sickness (MOSSick)
Source: Compr Psychoneuroendocrinol. 2025 Nov 13;24:100327. doi: 10.1016/j.cpnec.2025.100327 (PMC12666512; doi:10.1016/j.cpnec.2025.100327)
Supplement: Multimedia component 1 [file mmc1.docx]

**Skarp et al**

**The motivational drives of sickness: Acute changes in self-rated motivation during experimental endotoxemia using the the Motivation Scale of Sickness (MOSSick)**

**SUPPLEMENTARY MATERIAL**

**Supplementary text – R code**

#Setup--------------------------------------------------------------------------

# Clear workspace

rm(list=ls()) #Clear memory

graphics.off() #Clear plots

cat("\014") #Clear console

#install.packages("tidyverse")

#install.packages("geepack")

#install.packages("dplyr")

#install.packages("ggeffects")

#install.packages("multgee")

#install.packages("ggplot2")

#install.packages("Rmisc")

#install.packages("Cairo")

#install.packages("scales")

#install.packages("DescTools")

#install.packages("stringr")

#install.packages("repolr")

#install.packages("Rcpp") # for use with repolr

#install.packages("RcppArmadillo")

#tidyverse packages

library(tidyverse)

library(readxl)

library(dplyr)

#

library(multgee)

library(geepack)

library(DescTools)

library(stringr)

# Set working directory

setwd("C:/XXXX/")

#Read data file with MOSSick 23 and 25 as categories

df <- read_xlsx("C:/XXX.xlsx")

#Attach dataset

attach(df)

#Statistics GEE ----------------------------------------------------------------

## all items are reversed to have the effect of LPS/time on motivation in the right direction (higher = more likely)

##The MOSSick numbers below refer to the item numbers in the scale (e.g. MOSSick_1 = how hungry are you)

##MOSSick_1

#reverse answers for GEE

df <- df %>%

mutate(MOSSick_1 = case_when(

(MOSSick_1 == 1) ~ (6-MOSSick_1), (MOSSick_1 == 2) ~ (6-MOSSick_1),

(MOSSick_1 == 3) ~ (6-MOSSick_1), (MOSSick_1 == 4) ~ (6-MOSSick_1),

(MOSSick_1 == 5) ~ (6-MOSSick_1)

))

#GEE time.exch

library(multgee)

OLgee1 <- ordLORgee(formula=MOSSick_1~factor(Condition)*factor(Time), data = df,

id= newID, LORstr= "time.exch", link = "logit",

repeated = interaction(Day, Time))

summary(OLgee1)

CI_GEE_1 <- confint(OLgee1)

##MOSSick_2

#reverse answers for GEE

df <- df %>%

mutate(MOSSick_2 = case_when(

(MOSSick_2 == 1) ~ (6-MOSSick_2), (MOSSick_2 == 2) ~ (6-MOSSick_2),

(MOSSick_2 == 3) ~ (6-MOSSick_2), (MOSSick_2 == 4) ~ (6-MOSSick_2),

(MOSSick_2 == 5) ~ (6-MOSSick_2)

))

#GEE time.exch

library(multgee)

OLgee2 <- ordLORgee(formula=MOSSick_2~factor(Condition)*factor(Time), data = df,

id= newID, LORstr= "time.exch", link = "logit",

repeated = interaction(Day, Time))

summary(OLgee2)

CI_GEE_2 <- confint(OLgee2)

##MOSSick_3

#reverse answers for GEE

df <- df %>%

mutate(MOSSick_3 = case_when(

(MOSSick_3 == 1) ~ (6-MOSSick_3), (MOSSick_3 == 2) ~ (6-MOSSick_3),

(MOSSick_3 == 3) ~ (6-MOSSick_3), (MOSSick_3 == 4) ~ (6-MOSSick_3),

(MOSSick_3 == 5) ~ (6-MOSSick_3)

))

#GEE time.exch

library(multgee)

OLgee3 <- ordLORgee(formula=MOSSick_3~factor(Condition)*factor(Time), data = df,

id= newID, LORstr= "time.exch", link = "logit",

repeated = interaction(Day, Time))

summary(OLgee3)

CI_GEE_3 <- confint(OLgee3)

##MOSSick_4

#reverse answers for GEE

df <- df %>%

mutate(MOSSick_4 = case_when(

(MOSSick_4 == 1) ~ (6-MOSSick_4), (MOSSick_4 == 2) ~ (6-MOSSick_4),

(MOSSick_4 == 3) ~ (6-MOSSick_4), (MOSSick_4 == 4) ~ (6-MOSSick_4),

(MOSSick_4 == 5) ~ (6-MOSSick_4)

))

#GEE time.exch

library(multgee)

OLgee4 <- ordLORgee(formula=MOSSick_4~factor(Condition)*factor(Time), data = df,

id= newID, LORstr= "time.exch", link = "logit",

repeated = interaction(Day, Time))

summary(OLgee4)

CI_GEE_4 <- confint(OLgee4)

##MOSSick_5

#reverse answers for GEE

df <- df %>%

mutate(MOSSick_5 = case_when(

(MOSSick_5 == 1) ~ (6-MOSSick_5), (MOSSick_5 == 2) ~ (6-MOSSick_5),

(MOSSick_5 == 3) ~ (6-MOSSick_5), (MOSSick_5 == 4) ~ (6-MOSSick_5),

(MOSSick_5 == 5) ~ (6-MOSSick_5)

))

#GEE time.exch

library(multgee)

OLgee5 <- ordLORgee(formula=MOSSick_5~factor(Condition)*factor(Time), data = df,

id= newID, LORstr= "time.exch", link = "logit",

repeated = interaction(Day, Time))

summary(OLgee5)

CI_GEE_5 <- confint(OLgee5)

##MOSSick_6

#reverse answers for GEE

df <- df %>%

mutate(MOSSick_6 = case_when(

(MOSSick_6 == 1) ~ (6-MOSSick_6), (MOSSick_6 == 2) ~ (6-MOSSick_6),

(MOSSick_6 == 3) ~ (6-MOSSick_6), (MOSSick_6 == 4) ~ (6-MOSSick_6),

(MOSSick_6 == 5) ~ (6-MOSSick_6)

))

#GEE time.exch

library(multgee)

OLgee6 <- ordLORgee(formula=MOSSick_6~factor(Condition)*factor(Time), data = df,

id= newID, LORstr= "time.exch", link = "logit",

repeated = interaction(Day, Time))

summary(OLgee6)

CI_GEE_6 <- confint(OLgee6)

##MOSSick_7

#reverse answers for GEE

df <- df %>%

mutate(MOSSick_7 = case_when(

(MOSSick_7 == 1) ~ (6-MOSSick_7), (MOSSick_7 == 2) ~ (6-MOSSick_7),

(MOSSick_7 == 3) ~ (6-MOSSick_7), (MOSSick_7 == 4) ~ (6-MOSSick_7),

(MOSSick_7 == 5) ~ (6-MOSSick_7)

))

#GEE time.exch

library(multgee)

OLgee7 <- ordLORgee(formula=MOSSick_7~factor(Condition)*factor(Time), data = df,

id= newID, LORstr= "time.exch", link = "logit",

repeated = interaction(Day, Time))

summary(OLgee7)

CI_GEE_7 <- confint(OLgee7)

##MOSSick_8

#reverse answers for GEE

df <- df %>%

mutate(MOSSick_8 = case_when(

(MOSSick_8 == 1) ~ (6-MOSSick_8), (MOSSick_8 == 2) ~ (6-MOSSick_8),

(MOSSick_8 == 3) ~ (6-MOSSick_8), (MOSSick_8 == 4) ~ (6-MOSSick_8),

(MOSSick_8 == 5) ~ (6-MOSSick_8)

))

#GEE time.exch

library(multgee)

OLgee8 <- ordLORgee(formula=MOSSick_8~factor(Condition)*factor(Time), data = df,

id= newID, LORstr= "time.exch", link = "logit",

repeated = interaction(Day, Time))

summary(OLgee8)

CI_GEE_8 <- confint(OLgee8)

##MOSSick_9

#reverse answers for GEE

df <- df %>%

mutate(MOSSick_9 = case_when(

(MOSSick_9 == 1) ~ (6-MOSSick_9), (MOSSick_9 == 2) ~ (6-MOSSick_9),

(MOSSick_9 == 3) ~ (6-MOSSick_9), (MOSSick_9 == 4) ~ (6-MOSSick_9),

(MOSSick_9 == 5) ~ (6-MOSSick_9)

))

#GEE time.exch

library(multgee)

OLgee9 <- ordLORgee(formula=MOSSick_9~factor(Condition)*factor(Time), data = df,

id= newID, LORstr= "time.exch", link = "logit",

repeated = interaction(Day, Time))

summary(OLgee9)

CI_GEE_9 <- confint(OLgee9)

##MOSSick_10

#reverse answers for GEE

df <- df %>%

mutate(MOSSick_10 = case_when(

(MOSSick_10 == 1) ~ (6-MOSSick_10), (MOSSick_10 == 2) ~ (6-MOSSick_10),

(MOSSick_10 == 3) ~ (6-MOSSick_10), (MOSSick_10 == 4) ~ (6-MOSSick_10),

(MOSSick_10 == 5) ~ (6-MOSSick_10)

))

#GEE time.exch

library(multgee)

OLgee10 <- ordLORgee(formula=MOSSick_10~factor(Condition)*factor(Time), data = df,

id= newID, LORstr= "time.exch", link = "logit",

repeated = interaction(Day, Time))

summary(OLgee10)

CI_GEE_10 <- confint(OLgee10)

##MOSSick_11

#reverse answers for GEE

df <- df %>%

mutate(MOSSick_11 = case_when(

(MOSSick_11 == 1) ~ (6-MOSSick_11), (MOSSick_11 == 2) ~ (6-MOSSick_11),

(MOSSick_11 == 3) ~ (6-MOSSick_11), (MOSSick_11 == 4) ~ (6-MOSSick_11),

(MOSSick_11 == 5) ~ (6-MOSSick_11)

))

#GEE time.exch

library(multgee)

OLgee11 <- ordLORgee(formula=MOSSick_11~factor(Condition)*factor(Time), data = df,

id= newID, LORstr= "time.exch", link = "logit",

repeated = interaction(Day, Time))

summary(OLgee11)

CI_GEE_11 <- confint(OLgee11)

##MOSSick_12

#reverse answers for GEE

df <- df %>%

mutate(MOSSick_12 = case_when(

(MOSSick_12 == 1) ~ (6-MOSSick_12), (MOSSick_12 == 2) ~ (6-MOSSick_12),

(MOSSick_12 == 3) ~ (6-MOSSick_12), (MOSSick_12 == 4) ~ (6-MOSSick_12),

(MOSSick_12 == 5) ~ (6-MOSSick_12)

))

#GEE time.exch

library(multgee)

OLgee12 <- ordLORgee(formula=MOSSick_12~factor(Condition)*factor(Time), data = df,

id= newID, LORstr= "time.exch", link = "logit",

repeated = interaction(Day, Time))

summary(OLgee12)

CI_GEE_12 <- confint(OLgee12)

##MOSSick_13

#reverse answers for GEE

df <- df %>%

mutate(MOSSick_13 = case_when(

(MOSSick_13 == 1) ~ (6-MOSSick_13), (MOSSick_13 == 2) ~ (6-MOSSick_13),

(MOSSick_13 == 3) ~ (6-MOSSick_13), (MOSSick_13 == 4) ~ (6-MOSSick_13),

(MOSSick_13 == 5) ~ (6-MOSSick_13)

))

#GEE time.exch

library(multgee)

OLgee13 <- ordLORgee(formula=MOSSick_13~factor(Condition)*factor(Time), data = df,

id= newID, LORstr= "time.exch", link = "logit",

repeated = interaction(Day, Time))

summary(OLgee13)

CI_GEE_13 <- confint(OLgee13)

##MOSSick_14

#reverse answers for GEE

df <- df %>%

mutate(MOSSick_14 = case_when(

(MOSSick_14 == 1) ~ (6-MOSSick_14), (MOSSick_14 == 2) ~ (6-MOSSick_14),

(MOSSick_14 == 3) ~ (6-MOSSick_14), (MOSSick_14 == 4) ~ (6-MOSSick_14),

(MOSSick_14 == 5) ~ (6-MOSSick_14)

))

#GEE time.exch

library(multgee)

OLgee14 <- ordLORgee(formula=MOSSick_14~factor(Condition)*factor(Time), data = df,

id= newID, LORstr= "time.exch", link = "logit",

repeated = interaction(Day, Time))

summary(OLgee14)

CI_GEE_14 <- confint(OLgee14)

##MOSSick_15

#reverse answers for GEE

df <- df %>%

mutate(MOSSick_15 = case_when(

(MOSSick_15 == 1) ~ (6-MOSSick_15), (MOSSick_15 == 2) ~ (6-MOSSick_15),

(MOSSick_15 == 3) ~ (6-MOSSick_15), (MOSSick_15 == 4) ~ (6-MOSSick_15),

(MOSSick_15 == 5) ~ (6-MOSSick_15)

))

#GEE time.exch

library(multgee)

OLgee15 <- ordLORgee(formula=MOSSick_15~factor(Condition)*factor(Time), data = df,

id= newID, LORstr= "time.exch", link = "logit",

repeated = interaction(Day, Time))

summary(OLgee15)

CI_GEE_15 <- confint(OLgee15)

##MOSSick_16

#reverse answers for GEE

df <- df %>%

mutate(MOSSick_16 = case_when(

(MOSSick_16 == 1) ~ (6-MOSSick_16), (MOSSick_16 == 2) ~ (6-MOSSick_16),

(MOSSick_16 == 3) ~ (6-MOSSick_16), (MOSSick_16 == 4) ~ (6-MOSSick_16),

(MOSSick_16 == 5) ~ (6-MOSSick_16)

))

#GEE time.exch

library(multgee)

OLgee16 <- ordLORgee(formula=MOSSick_16~factor(Condition)*factor(Time), data = df,

id= newID, LORstr= "time.exch", link = "logit",

repeated = interaction(Day, Time))

summary(OLgee16)

CI_GEE_16 <- confint(OLgee16)

##MOSSick_17

#reverse answers for GEE

df <- df %>%

mutate(MOSSick_17 = case_when(

(MOSSick_17 == 1) ~ (6-MOSSick_17), (MOSSick_17 == 2) ~ (6-MOSSick_17),

(MOSSick_17 == 3) ~ (6-MOSSick_17), (MOSSick_17 == 4) ~ (6-MOSSick_17),

(MOSSick_17 == 5) ~ (6-MOSSick_17)

))

#GEE time.exch

library(multgee)

OLgee17 <- ordLORgee(formula=MOSSick_17~factor(Condition)*factor(Time), data = df,

id= newID, LORstr= "time.exch", link = "logit",

repeated = interaction(Day, Time))

summary(OLgee17)

CI_GEE_17 <- confint(OLgee17)

##MOSSick_18

#reverse answers for GEE

df <- df %>%

mutate(MOSSick_18 = case_when(

(MOSSick_18 == 1) ~ (6-MOSSick_18), (MOSSick_18 == 2) ~ (6-MOSSick_18),

(MOSSick_18 == 3) ~ (6-MOSSick_18), (MOSSick_18 == 4) ~ (6-MOSSick_18),

(MOSSick_18 == 5) ~ (6-MOSSick_18)

))

#GEE time.exch

library(multgee)

OLgee18 <- ordLORgee(formula=MOSSick_18~factor(Condition)*factor(Time), data = df,

id= newID, LORstr= "time.exch", link = "logit",

repeated = interaction(Day, Time))

summary(OLgee18)

CI_GEE_18 <- confint(OLgee18)

##MOSSick_19

#reverse answers for GEE

df <- df %>%

mutate(MOSSick_19 = case_when(

(MOSSick_19 == 1) ~ (6-MOSSick_19), (MOSSick_19 == 2) ~ (6-MOSSick_19),

(MOSSick_19 == 3) ~ (6-MOSSick_19), (MOSSick_19 == 4) ~ (6-MOSSick_19),

(MOSSick_19 == 5) ~ (6-MOSSick_19)

))

#GEE time.exch

library(multgee)

OLgee19 <- ordLORgee(formula=MOSSick_19~factor(Condition)*factor(Time), data = df,

id= newID, LORstr= "time.exch", link = "logit",

repeated = interaction(Day, Time))

summary(OLgee19)

CI_GEE_19 <- confint(OLgee19)

##MOSSick_20

#reverse answers for GEE

df <- df %>%

mutate(MOSSick_20 = case_when(

(MOSSick_20 == 1) ~ (6-MOSSick_20), (MOSSick_20 == 2) ~ (6-MOSSick_20),

(MOSSick_20 == 3) ~ (6-MOSSick_20), (MOSSick_20 == 4) ~ (6-MOSSick_20),

(MOSSick_20 == 5) ~ (6-MOSSick_20)

))

#GEE time.exch

library(multgee)

OLgee20 <- ordLORgee(formula=MOSSick_20~factor(Condition)*factor(Time), data = df,

id= newID, LORstr= "time.exch", link = "logit",

repeated = interaction(Day, Time))

summary(OLgee20)

CI_GEE_20 <- confint(OLgee20)

##MOSSick_21

#reverse answers for GEE

df <- df %>%

mutate(MOSSick_21 = case_when(

(MOSSick_21 == 1) ~ (6-MOSSick_21), (MOSSick_21 == 2) ~ (6-MOSSick_21),

(MOSSick_21 == 3) ~ (6-MOSSick_21), (MOSSick_21 == 4) ~ (6-MOSSick_21),

(MOSSick_21 == 5) ~ (6-MOSSick_21)

))

#GEE time.exch

library(multgee)

OLgee21 <- ordLORgee(formula=MOSSick_21~factor(Condition)*factor(Time), data = df,

id= newID, LORstr= "time.exch", link = "logit",

repeated = interaction(Day, Time))

summary(OLgee21)

CI_GEE_21 <- confint(OLgee21)

##MOSSick_22

#reverse answers for GEE

df <- df %>%

mutate(MOSSick_22 = case_when(

(MOSSick_22 == 1) ~ (6-MOSSick_22), (MOSSick_22 == 2) ~ (6-MOSSick_22),

(MOSSick_22 == 3) ~ (6-MOSSick_22), (MOSSick_22 == 4) ~ (6-MOSSick_22),

(MOSSick_22 == 5) ~ (6-MOSSick_22)

))

#GEE time.exch

library(multgee)

OLgee22 <- ordLORgee(formula=MOSSick_22~factor(Condition)*factor(Time), data = df,

id= newID, LORstr= "time.exch", link = "logit",

repeated = interaction(Day, Time))

summary(OLgee22)

CI_GEE_22 <- confint(OLgee22)

#GEE uniform

library(multgee)

OLgee22b <- ordLORgee(formula=MOSSick_22~factor(Condition)*factor(Time), data = df,

id= newID, LORstr= "uniform", link = "logit",

repeated = interaction(Day, Time))

summary(OLgee22b)

CI_GEE_22b <- confint(OLgee22b)

##MOSSick_23 (Categorical)

#reverse answers for GEE

df <- df %>%

mutate(MOSSick_23_categ = case_when(

(MOSSick_23_categ == 1) ~ (6-MOSSick_23_categ), (MOSSick_23_categ == 2) ~ (6-MOSSick_23_categ),

(MOSSick_23_categ == 3) ~ (6-MOSSick_23_categ), (MOSSick_23_categ == 4) ~ (6-MOSSick_23_categ),

(MOSSick_23_categ == 5) ~ (6-MOSSick_23_categ)

))

#GEE time.exch

library(multgee)

OLgee23 <- ordLORgee(formula=MOSSick_23_categ~factor(Condition)*factor(Time), data = df,

id= newID, LORstr= "time.exch", link = "logit",

repeated = interaction(Day, Time))

summary(OLgee23)

CI_GEE_23 <- confint(OLgee23)

##MOSSick_25 (categorical)

#reverse answers for GEE

df <- df %>%

mutate(MOSSick_25_categ = case_when(

(MOSSick_25_categ == 1) ~ (6-MOSSick_25_categ), (MOSSick_25_categ == 2) ~ (6-MOSSick_25_categ),

(MOSSick_25_categ == 3) ~ (6-MOSSick_25_categ), (MOSSick_25_categ == 4) ~ (6-MOSSick_25_categ),

(MOSSick_25_categ == 5) ~ (6-MOSSick_25_categ)

))

#GEE time.exch

library(multgee)

OLgee25 <- ordLORgee(formula=MOSSick_25_categ~factor(Condition)*factor(Time), data = df,

id= newID, LORstr= "time.exch", link = "logit",

repeated = interaction(Day, Time))

summary(OLgee25)

CI_GEE_25 <- confint(OLgee25)

#------------------------

#POST-HOC GEE's FOR ITEMS WITH SIGNIFICANT RESULTS IN BOTH CONDITIONS ----------

#are LPS-induced changes significant?

##MOSSick 1, 14, 13, 20, 22,

#Subset dataframe with only LPS.

dfLPS <- subset(df, Condition == "1")

#MOSSick1.

OLgee1L <- ordLORgee(formula=MOSSick_1~factor(Time), data = dfLPS,

id= newID, LORstr= "time.exch", link = "logit",

repeated = interaction (Day, Time))

summary(OLgee1L)

CI_GEE_1L <- confint(OLgee1L)

#MOSSick14

OLgee14L <- ordLORgee(formula=MOSSick_14~factor(Time), data = dfLPS,

id= newID, LORstr= "time.exch", link = "logit",

repeated = interaction (Day, Time))

summary(OLgee14L)

CI_GEE_14L <- confint(OLgee14L)

#MOSSick13

OLgee13L <- ordLORgee(formula=MOSSick_13~factor(Time), data = dfLPS,

id= newID, LORstr= "time.exch", link = "logit",

repeated = interaction (Day, Time))

summary(OLgee13L)

CI_GEE_13L <- confint(OLgee13L)

#MOSSick20

OLgee20L <- ordLORgee(formula=MOSSick_20~factor(Time), data = dfLPS,

id= newID, LORstr= "time.exch", link = "logit",

repeated = interaction (Day, Time))

summary(OLgee20L)

CI_GEE_20L <- confint(OLgee20L)

#MOSSick22

OLgee22L <- ordLORgee(formula=MOSSick_22~factor(Time), data = dfLPS,

id= newID, LORstr= "time.exch", link = "logit",

repeated = interaction (Day, Time))

summary(OLgee22L)

CI_GEE_22L <- confint(OLgee22L)

#Does not converge

OLgee22L <- ordLORgee(formula=MOSSick_22~factor(Time), data = dfLPS,

id= newID, LORstr= "uniform", link = "logit",

repeated = interaction (Day, Time))

summary(OLgee22L)

CI_GEE_22L <- confint(OLgee22L)

#Correlation pre-processing ----------------------------------------------------

#Read data file with Delta value

dfDelta <- read_xlsx("C:/XXXX.xlsx")

#Attach dataset

detach(df)

attach(dfDelta)

#Kendall's Correlation between category and SQ, BT, and IL-6--------------------------

#SQ

cor.test(Hunger, T3_SicknessQTOT, method = "kendall")

cor.test(Food, T3_SicknessQTOT, method = "kendall")

cor.test(Physical, T3_SicknessQTOT, method = "kendall")

cor.test(Rest, T3_SicknessQTOT, method = "kendall")

cor.test(Social, T3_SicknessQTOT, method = "kendall")

cor.test(Care, T3_SicknessQTOT, method = "kendall")

cor.test(Pay, T3_SicknessQTOT, method = "kendall")

#BT

cor.test(Hunger, T3_temp, method = "kendall")

cor.test(Food, T3_temp, method = "kendall")

cor.test(Physical, T3_temp, method = "kendall")

cor.test(Rest, T3_temp, method = "kendall")

cor.test(Social, T3_temp, method = "kendall")

cor.test(Care, T3_temp, method = "kendall")

cor.test(Pay, T3_temp, method = "kendall")

#IL6

cor.test(Hunger, T3_IL6, method = "kendall")

cor.test(Food, T3_IL6, method = "kendall")

cor.test(Physical, T3_IL6, method = "kendall")

cor.test(Rest, T3_IL6, method = "kendall")

cor.test(Social, T3_IL6, method = "kendall")

cor.test(Care, T3_IL6, method = "kendall")

cor.test(Pay, T3_IL6, method = "kendall")

#TNFa

cor.test(Hunger, T3_TNFa, method = "kendall")

cor.test(Food, T3_TNFa, method = "kendall")

cor.test(Physical, T3_TNFa, method = "kendall")

cor.test(Rest, T3_TNFa, method = "kendall")

cor.test(Social, T3_TNFa, method = "kendall")

cor.test(Care, T3_TNFa, method = "kendall")

cor.test(Pay, T3_TNFa, method = "kendall")

##SENSITIVITY ANALYSIS: correlation w/o IL6/TNFa outlier

##Correlation pre-processing ----------------------------------------------------

#Read data file with Delta value w/o outlier

dfDelta2 <- read_xlsx("C:/XXXXX.xlsx")

#Attach dataset

detach(dfDelta)

attach(dfDelta2)

#Kendall's Correlation between category and SQ, BT, and IL-6--------------------------

#IL6

cor.test(Hunger, T3_IL6, method = "kendall")

cor.test(Food, T3_IL6, method = "kendall")

cor.test(Physical, T3_IL6, method = "kendall")

cor.test(Rest, T3_IL6, method = "kendall")

cor.test(Social, T3_IL6, method = "kendall")

cor.test(Care, T3_IL6, method = "kendall")

cor.test(Pay, T3_IL6, method = "kendall")

#TNFa

cor.test(Hunger, T3_TNFa, method = "kendall")

cor.test(Food, T3_TNFa, method = "kendall")

cor.test(Physical, T3_TNFa, method = "kendall")

cor.test(Rest, T3_TNFa, method = "kendall")

cor.test(Social, T3_TNFa, method = "kendall")

cor.test(Care, T3_TNFa, method = "kendall")

cor.test(Pay, T3_TNFa, method = "kendall")

##SENSITIVITY ANALYSIS: correlation SQ w/o motiv items (1, 3, 4)

##Correlation pre-processing ----------------------------------------------------

#Read data file with Delta value

dfDelta3 <- read_xlsx("C:/XXXXXX.xlsx")

#Attach dataset

detach(dfDelta2)

attach(dfDelta3)

#Kendall's Correlation between category and SQ--------------------------

#SQ

cor.test(Hunger, T3_SicknessQ_Tot_woMotiv, method = "kendall")

cor.test(Food, T3_SicknessQ_Tot_woMotiv, method = "kendall")

cor.test(Physical, T3_SicknessQ_Tot_woMotiv, method = "kendall")

cor.test(Rest, T3_SicknessQ_Tot_woMotiv, method = "kendall")

cor.test(Social, T3_SicknessQ_Tot_woMotiv, method = "kendall")

cor.test(Care, T3_SicknessQ_Tot_woMotiv, method = "kendall")

cor.test(Pay, T3_SicknessQ_Tot_woMotiv, method = "kendall")

**Table S1 – Associations between motivational changes during sickness and intensity of the sickness response 3 hours post-LPS injection.**

|  | **Hunger** | **Food** | **Physical** | **Rest** | **Social** | **Care** | **Pay** |
| --- | --- | --- | --- | --- | --- | --- | --- |
| SicknessQ | τ=-0.08 p=0.64 | τ=-0.19 p=0.27 | τ=-0.19 p=0.25 | τ=0.35 p=0.04 | τ=-0.36 p=0.03 | τ=0.19 p=0.25 | τ=0.24 p=0.16 |
| SicknessQ without motivational items | τ=-0.07 p=0.69 | τ=-0.08 p=0.64 | τ=-0.11  p=0.50 | τ=0.22 p=0.19 | τ=-0.33 p=0.048 | τ=0.15 p=0.37 | τ=0.22 p=0.19 |
| Temperature | τ=-0.04 p=0.81 | τ=0.02 p=0.93 | τ=0.06 p=0.71 | τ=0.18 p=0.27 | τ=-0.26 p=0.12 | τ=0.06 p=0.71 | τ=-0.01 p=0.95 |
| IL-6 | τ=0.18 p=0.27 | τ=0.14 p=0.39 | τ=-0.13 p=0.44 | τ=0.16 p=0.33 | τ=-0.28 p=0.08 | τ=-0.004 p=0.98 | τ=-0.04 p=0.83 |
| TNF-α | τ=-0.15 p=0.36 | τ=-0.06 p=0.71 | τ=-0.12 p=0.48 | τ=0.05 p=0.76 | τ=-0.21 p=0.19 | τ=-0.02 p=0.88 | τ=-0.23 p=0.17 |

Kendall’s tau-b.

**Table S2 – Associations between motivational changes during sickness and IL-6 and TNF-α concentrations 3 hours post-LPS injection – excluding one outlier in cytokines.**

|  | **Hunger** | **Food** | **Physical** | **Rest** | **Social** | **Care** | **Pay** |
| --- | --- | --- | --- | --- | --- | --- | --- |
| IL-6 | τ=0.29 p=0.09 | τ=0.10 p=0.57 | τ=-0.14 p=0.41 | τ=0.14 p=0.40 | τ=-0.23 p=0.17 | τ=-0.04 p=0.82 | τ=0.05 p=0.77 |
| TNF-α | τ=-0.08 p=0.64 | τ=-0.13 p=0.45 | τ=-0.13 p=0.45 | τ=0.02 p=0.90 | τ=-0.15 p=0.36 | τ=-0.06 p=0.72 | τ=-0.16 p=0.34 |

Kendall’s tau-b.
